# Association of self-reported physical activity patterns and socio-demographic factors among normal-weight and overweight Japanese men

**DOI:** 10.1186/1471-2458-12-278

**Published:** 2012-05-21

**Authors:** Yung Liao, Kazuhiro Harada, Ai Shibata, Kaori Ishii, Koichiro Oka, Yoshio Nakamura, Shigeru Inoue, Teruichi Shimomitsu

**Affiliations:** 1 Graduate School of Sport Sciences, Waseda University, 2-579-15 Mikajima, Tokorozawa, Saitama, 359-1192, Japan; 2 Japan Society for the Promotion of Science, Sumitomo-Ichibancho Bldg., 6 Ichibancho, Chiyoda-ku, Tokyo, 102-8471, Japan; 3 Faculty of Sport Sciences, Waseda University, 2-579-15 Mikajima, Tokorozawa, Saitama, 359-1192, Japan; 4 Department of Preventive Medicine and Public Health, Tokyo Medical University, 6-1-1 Shinjuku, Shinjuku-ku, Tokyo, 1608402, Japan

**Keywords:** Physical activity patterns, Socio-demographic factors, Overweight, BMI, Japanese

## Abstract

**Background:**

It is still not known whether overweight men have different patterns and socio-demographic correlates of self-reported physical activity (PA) compared with normal-weight men. Thus, this study examined the perceived PA patterns and associated socio-demographic factors among normal-weight and overweight Japanese men.

**Methods:**

Data were analyzed for 1,420 men (aged 44.48.3years) who responded to an Internet-based cross-sectional survey relating to socio-demographic variables, BMI status, and a short version of the International Physical Activity Questionnaire. Mann-Whitney, chi-square, and binary logistic regression analyses were employed.

**Results:**

Normal-weight men were significantly more likely to attain 150 minutes per week of moderate-to-vigorous PA than overweight men (26.6% vs. 21.3%; *p*=0.035), whereas there were no significant proportional differences in total PA and walking between the two BMI subgroups. With PA, a significant interaction was observed between BMI status and household income (*p*=0.004 for total PA; *p*=0.02 for walking). In the subgroup analyses, having a lower household income (odds ratio, 0.63; 95% confidence interval, 0.41-0.96) was negatively associated with attaining 150 minutes of walking per week among normal-weight men. No significant associations between household income and attaining 150 minutes per week of total PA and walking were found among overweight men.

**Conclusions:**

The results revealed that patterns and socio-demographic correlates of self-reported PA in overweight men are different from those in normal-weight men. This finding suggests the necessity of developing specific strategies for PA intervention among overweight men. Socio-demographic correlates of PA may be more important for normal-weight than overweight men.

## Background

Obesity is an important risk factor in cardiovascular diseases, type 2 diabetes, cancer, hypertension, and premature death as well as in psychosocial problems [1,2]. Despite such deleterious health outcomes, an increasing prevalence of being overweight and obese has been reported around the world, and if the present trend continues, 57.8% of the worlds adult population is predicted to be either overweight or obese by 2030 [3]. This trend has also been observed in Japan: in 2009, 30.5% of men and 20.8% of women were overweight or obese, with men aged 40-49years accounting for the highest percentage (36.2%) [4]. Thus, in Japan, developing effective strategies and programs for the prevention and treatment of overweight and obese individuals, especially middle-aged men, should be considered an urgent need in public health.

It has been well documented that physical activity (PA) contributes to primary and secondary prevention of several chronic diseases, and in addition it has a dose-response effect on obesity [5,6]. As a result, promoting PA for overweight or obese adults should be a public-health concern. In designing relevant policies and effective interventions for obesity prevention, a better understanding of the patterns and socio-demographic correlates of PA among overweight or obese groups is critical because this information could help in targeting socio-demographic subgroups that are less likely to engage in sufficient PA. A large number of studies have examined PA patterns [7,8] and socio-demographic factors associated with PA among different populations and inactive subgroups [9-11]. Regarding overweight and obese populations, some studies observed that PA patterns differed according to weight status [12-14]. Similarly, a study conducted on Brazilian adults indicated that socio-demographic variables (e.g., sex and education) associated with self-reported PA seem to differ between normal-weight and overweight or obese adults [15]. The study suggested that this was due to overweight and obese individuals having different motivations or preferences with respect to PA participation compared with normal-weight people.

The importance of further identifying how patterns of PA and socio-demographic characteristics differ according to BMI status was highlighted in these previous studies in order to emphasize the effect of PA intervention for overweight and obese adults [12-15]. However, to date, few studies have examined PA patterns and associated socio-demographic factors stratified by BMI status. Substantial evidence on this issue is necessary for intervention designers and policy makers, especially in Asia, which has faced a growing obesity epidemic in recent years. In addition, previous studies have compared only the time spent in PA of different intensities (e.g., moderate-, vigorous-, or moderate- to vigorous-intensity activities) between normal and overweight or obese individuals [12-14]. However, few studies have described and compared the behavioral domains (e.g., walking, other type of PA) between normal and overweight or obese people. The aim of the present study was to determine whether there should be a focus on a particular domain of PA behavior and also whether special consideration should be given to individuals most in need of intervention when designing effective PA programs for overweight Japanese men. Thus, the present study examined the self-reported PA patterns and associated socio-demographic factors among Japanese normal-weight and overweight men.

## Methods

### Participants

An Internet-based cross-sectional survey was conducted in January 2009 by a Japanese Internet-research service company, which holds data, including socio-demographic attributes, on approximately 264,000 adult registrants. This allows the company to target a particular demographic group according to a surveys requirements. The present study sought a sample of approximately 3,000 adults aged between 30 and 59years, with 500 men and 500 women from three age-groups (30-39, 40-49, and 50-59years). In all, 9,418 adults were randomly selected from the database and received an e-mail inviting them to participate in this Internet-based survey. The Internet-research service company offered rewards points valued at 40 yen (one US dollar was equivalent to approximately 109 yen at the time). Of the selected adults, 3,000 individuals answered the survey questions online (response rate: 31.9%). The study received prior approval from the Ethics Committee of the Faculty of Sports Sciences, Waseda University, Japan.

### Socio-demographic variables

In the present study, the socio-demographic variables obtained from the research company included gender, age (categorized as 30-39, 40-49, and 50-59years), marital status (classified as married and unmarried), educational level (categorized as junior high and high school graduation, two years college degree or equivalent, and four years college or higher degree), job status (classified as full-time and not full-time employment), and household income (categorized as less than 5 million yen, 5-10 million yen, and over 10 million yen).

### Self-reported PA measures

PA was measured by the self-administered, short version of the International Physical Activity Questionnaire (IPAQ-SV), which has been recommended for national prevalence studies [16]. The IPAQ-SV, which includes seven items, was used to measure the frequency and duration of vigorous-intensity PA, moderate-intensity PA, and walking-level PA for young and middle-aged adults (15-69years). The test-retest reliability (r=0.72-0.93) and criterion validity (r=0.39) of the Japanese version of the IPAQ-SV are good and acceptable, respectively [17]. The total number of minutes per week in each PA category was computed. In the present study, three outcome variables in the IPAQ-SV were calculated: (1) total PAthe total number of minutes spent walking and undergoing moderate and vigorous activity; (2) walkingthe total number of minutes spent walking; (3) moderate-to-vigorous PA (MVPA)the total number of minutes engaged in moderate-to-vigorous activity. The three outcome variables were dichotomized into <150 minutes and 150 minutes according to public-health PA recommendations [6].

### Body mass index measures

Self-reported height and weight were utilized to calculate the body mass index (BMI; body weight in kilograms divided by the square of the height in meters). Participants with a BMI 25kg/m^2^ were defined as overweight in the present study.

### Data analysis

The data were analyzed from 1,420 men who provided complete information for the study variables. All analyses were stratified by BMI status. Mann-Whitney and chi-square test analyses were used to compare the differences in the time spent in PA, identify sample characteristics, and determine the proportional differences in attaining a weekly total of 150 minutes of PA, walking, and MVPA between normal-weight and overweight men. In addition, forced-entry adjusted binary logistic regression was conducted to examine the association between socio-demographic factors and PA (separately for total PA, walking, and MVPA) in the total sample. Likelihood ratio tests were used to compare models with or without interaction terms between socio-demographic variables and BMI status for the three PA outcome variables. If a significant interaction was observed, the sample was categorized according to BMI status. Then, for the subgroup analyses, forced-entry binary logistic regression analyses were conducted to examine the association between socio-demographic factors and PA. Odds ratios (ORs) and 95% confidence intervals (CI) were calculated for each variable. Inferential statistics were performed using SPSS 18.0, and the level of significance was set at *p*<0.05.

## Results

### Characteristics of respondents

Table1 presents the characteristics of the respondents (mean age, 44.48.3years). Of the respondents, 31.1% were overweight, 70.4% were married, 64.4% had an educational level of four years college or graduate school, 92.0% were in full-time employment, and 49.7% had a household income of between 5 million and 10 million yen. The prevalence of achieving 150 minutes per week of total PA was 57.4%.

**Table 1:** Characteristic of respondents

		BMI status		
	Total sample	Normal weight men	Overweight men	***p-value***
**Number (%)**	1,420	979 (68.9%)	441 (31.1%)	
**Age group**				0.18
30-39	475 (33.4%)	338 (34.5%)	137 (31.1%)	
40-49	474 (33.4%)	312 (31.9%)	162 (36.7%)	
50-59	471 (33.2%)	329 (33.6%)	142 (32.2%)	
**Marital status**				0.86
Married	1,000 (70.4%)	688 (70.3%)	312 (70.7%)	
Unmarried	420 (29.6%)	291 (29.7%)	129 (29.3%)	
**Educational level**				0.36
Junior high/ high school	330 (23.2%)	219 (22.4%)	111 (25.2%)	
2-year college	176 (12.4%)	118 (12.1%)	58 (13.2%)	
4-year college/ graduate school	914 (64.4%)	642 (65.5%)	272 (61.6%)	
**Job status**				0.77
Full-time job	1,306 (92.0%)	899 (91.8%)	407 (92.3%)	
Not full-time job	114 (8.0%)	80 (8.2%)	34 (7.7%)	
**Household income**				0.65
<5,000,000 yen	488 (34.4%)	343 (35.0%)	145 (32.9%)	
<10,000,000 yen	706 (49.7%)	481 (49.1%)	225 (51.0%)	
>10,000,000 yen	226 (15.9%)	155 (15.9%)	71 (16.1%)	
**Total PA**				0.16
150min +	815 (57.4%)	668 (58.6%)	241 (54.6%)	
<150min	605 (42.6%)	574 (41.4%)	200 (45.4%)	

### Differences in PA between normal-weight and overweight men

Table2 shows the mean reported time spent for total PA, walking, and MVPA as well as the categorical differences in attaining 150 minutes of total PA, walking, and MVPA between normal-weight and overweight men. No significant differences were found in the mean time spent in total PA (*p*=0.32) and walking (*p*=0.79) as well as in the proportion attaining 150 minutes of total PA (*p*=0.16) and walking (*p*=0.36) between the two BMI subgroups. However, significant differences were observed in the mean time spent engaged in MVPA (*p*=0.001) and in the proportion attaining 150 minutes of MVPA (*p*=0.035) between normal-weight and overweight men. The results revealed that normal-weight men were statistically significantly more likely to engage in MVPA and attain 150 minutes of MVPA than overweight men (26.6% vs. 21.3%, respectively).

**Table 2:** Mean time spent engaged in PA and the prevalence of attaining PA recommendations according to BMI status

		BMI status		
	Total	Normal-weight men	Overweight men	*p*-value
Mean time spent in total PA, min/week (SD)	330.7 (488.5)	340.4 (488.9)	306.2 (433.0)	0.32
Meet 150min of total PA per week (%)	57.4%	58.6%	54.6%	0.16
Mean time spent in walking, min/week (SD)	194.0 (302.1)	196.0 (308.4)	189.5 (288.0)	0.79
Meet 150min of walking per week (%)	41.8%	41.0%	43.5%	0.36
Mean time spent in MVPA, min/week (SD)	135.8 (298.9)	144.4 (315.5)	116.7 (257.7)	0.001*
Meet 150min of MVPA per week (%)	24.9%	26.6%	21.3%	0.035*

* Statistically significant (*p*<0.05).

SD=standard deviation.

### Socio-demographic correlates of attaining the PA recommendations in the total sample

The ORs for attaining 150 minutes of total PA, walking, and MVPA in the total sample are presented in Table3 according to age, marital status, educational level, job status, and household income. As Table3 shows, having a lower household income was negatively related to attaining 150 minutes of total PA (OR, 0.66; 95% CI, 0.46-0.94) and walking (OR, 0.67; 95% CI, 0.47-0.95). Table3 also shows that men not in full-time employment were more likely to engage in 150 minutes of MVPA (OR, 1.97; 95% CI, 1.26-3.06) than those in full-time employment.

**Table 3:** Socio-demographic correlates of attaining PA recommendations in the total sample

	150 minutes of total PA	150 minutes of walking	150 minutes of MVPA
	OR (95% CI)	OR (95% CI)	OR (95% CI)
**Age group**			
30-39	1.00 (ref.)	1.00 (ref.)	1.00 (ref.)
40-49	0.91 (0.70-1.18)	0.96 (0.74-1.25)	0.99 (0.73-1.34)
50-59	1.05 (0.80-1.39)	1.13 (0.86-1.50)	0.95 (0.70-1.31)
**Marital status**			
Unmarried	1.00 (ref.)	1.00 (ref.)	1.00 (ref.)
Married	1.06 (0.82-1.36)	1.26 (0.97-1.62)	0.92 (0.68-1.23)
**Educational level**			
4years of college/ graduate school	1.00 (ref.)	1.00 (ref.)	1.00 (ref.)
2years of college	1.03 (0.74-1.43)	1.08 (0.77-1.51)	1.11 (0.76-1.62)
Junior high/ high school	0.94 (0.72-1.23)	0.90 (0.69-1.18)	1.24 (0.92-1.68)
**Job status**			
Full-time job	1.00 (ref.)	1.00 (ref.)	1.00 (ref.)
Not full-time job	1.14 (0.75-1.73)	0.85 (0.56-1.31)	1.97 (1.26-3.06)*
**Household income**			
> 10,000,000 yen	1.00 (ref.)	1.00 (ref.)	1.00 (ref.)
< 10,000,000 yen	0.88 (0.64-1.21)	0.86 (0.63-1.17)	0.81 (0.57-1.14)
< 5,000,000 yen	0.66 (0.46-0.94)*	0.67 (0.47-0.95)*	0.71 (0.47-1.06)

* Statistically significant (*p*<0.05).

### Significance of interactions between BMI status and socio-demographic variables

Regarding total PA and walking, a significant interaction was observed between BMI status and household income (*p*=0.004 for total PA; *p*=0.02 for walking) (Table4). Therefore, subgroup analyses for the associations between socio-demographic correlates and PA were conducted among normal-weight and overweight men.

**Table 4:** Significance of interactions between BMI status and socio-demographic variables by binary logistic regression models

				*p*-value for interaction term with BMI status
	Total PA	Walking	MVPA (excluding walking)
Socio-demographic variables			
Age group (ref. 30-39years)			
40-49years	0.58	0.68	0.99
50-59years	0.81	0.37	0.49
Marital status (ref. Unmarried)			
Married	0.94	0.24	0.50
Educational level (ref. 4years college/graduate school)			
2years college	0.43	0.61	0.38
Junior high/high school	0.58	0.48	0.18
Job status (ref. Full-time job)			
Not full-time job	0.83	0.34	0.51
Household income (ref. > 10,000,000 yen)			
< 10,000,000 yen	0.76	0.83	0.68
< 5,000,000 yen	0.004*	0.02*	0.07

Adjusted by age, marital status, educational level, household income, employment status, and BMI status.

* Statistically significant (*p*<0.05)

### Associations between household income and attaining the PA recommendations among normal-weight and overweight men

The ORs for attaining 150 minutes of total PA and walking are presented in Table5 according to household income. As Table5 shows, no significant associations were observed between household income and attaining 150 minutes of total PA among both normal-weight and overweight men. For normal-weight men, lower household income was negatively related to attaining 150 minutes of walking (OR, 0.63; 95% CI, 0.41-0.96). No significant associations were found between household income and attaining 150 minutes of walking among overweight men.

**Table 5:** Associations between household income and attaining PA recommendations according to BMI status

	150 minutes of total PA		150 minutes of walking	
	Normal-weight Men	Overweight Men	Normal-weight Men	Overweight Men
	OR (95% CI)	OR (95% CI)	OR (95% CI)	OR (95% CI)
**Household income**				
> 10,000,000 yen	1.00 (ref.)	1.00 (ref.)	1.00 (ref.)	1.00 (ref.)
< 10,000,000 yen	0.76 (0.52-1.12)	1.19 (0.68-2.07)	0.76 (0.52-1.10)	1.14 (0.66-1.99)
< 5,000,000 yen	0.65 (0.43-1.01)	0.66 (0.35-1.26)	0.63 (0.41-0.96)*	0.76 (0.40-1.45)

* Statistically significant (*p*<0.05).

## Discussion

This study examined the patterns and socio-demographic correlates of self-reported PA among normal-weight and overweight men. The results revealed that both self-reported PA patterns and associated correlates in overweight men were different from those in normal-weight men, which is consistent with the findings of previous studies [15,18]. Our results imply that BMI status should be considered when developing more effective intervention approaches to PA among Japanese men.

With regard to the prevalence of attaining 150 minutes per week of total PA, walking, and MVPA, no significant differences were found in total PA and walking among the BMI subgroups; however, overweight men were significantly less likely to achieve 150 minutes of MVPA than normal-weight men. Numerous studies have reported that overweight or obese individuals spend less time on PA and are less likely than normal-weight individuals to meet the minimum recommended level of PA [13,14,19-21]. However, few studies have stratified PA time into walking and MVPA, yet this division provides a better understanding of the patterns of walking and MVPA when developing effective PA intervention strategies. Using objective PA measurements, one study showed that overweight or obese children had a distinct pattern of daily MVPA compared with normal-weight children [12]. Furthermore, behavioral preferences for sedentary behavior or light activity have been reported among overweight and obese individuals [22]. A possible explanation for the latter result is that owing to their poor physical condition, it may be more difficult for overweight men to engage in MVPA (e.g., leisure-time PA, sports, and vigorous types of recreational activities) compared with walking [12].

Regardless of weight status, men who had lower household income were less likely to attain 150 minutes of total PA and walking, which is consistent with previous findings [9,10]. In addition, men without a full-time job were more likely to achieve 150 minutes of MVPA. This finding has not been demonstrated in previous studies [9,10]. A possible explanation for this result is that job status may be related to whether men have available or limited opportunity to engage in MVPA (e.g., leisure-time PA, sports, and vigorous types of recreational activities) in their leisure time.

In logistic regression models, the interactions between BMI and five socio-demographic variables (age, marital status, educational level, job status, and household income) separately for the three PA outcome variables were tested based on likelihood ratio tests. Only household income was revealed as a different socio-demographic correlate of PA between normal-weight and overweight men. Consistent with the findings of a Brazilian study [15], we did not observe an interaction between BMI and age. In contrast, an interaction between BMI and education, which was identified in that study [15], was not observed in the present study. In addition, previous studies have indicated that adults with a higher household income were more likely to be physically active [9,10]. However, there has been no discussion or analysis as to whether this association differs according to BMI status. A possible mechanism underlying the observed significance in household income only among normal-weight men is that household income may not be a barrier or facilitator for overweight men to engage in walking compared with normal-weight men. Therefore, the findings of the present study suggest that correlates of specific PA may vary according to BMI status.

Furthermore, in the subgroup analyses, household income was significantly associated with achieving 150 minutes of walking among normal-weight men, whereas no significant associations between household income and attaining 150 minutes of walking were observed among overweight men. Consistent with the findings of a Brazilian study [15], this result implies that socio-demographic correlates are less important in overweight than in normal-weight men and that other correlates of meeting recommended levels of PA may be more important for overweight men, such as psychosocial correlates and environmental factors.

Based on the findings of the present study, encouraging overweight men to engage in walking could be considered a gateway for them to achieve health-enhancing levels of PA. More factors associated with walking or other specific MVPA behaviors need to be further identified to develop tailored PA for overweight men. In addition, for normal-weight men, the promotion of daily walking (e.g., walking for transportation or recreation) should be targeted at those with a lower household income. Therefore, it is expected that future studies will identify the multiple levels of correlates associated with specific types of PA among normal-weight and overweight men.

There were some limits with the current study. First, the main measurements of the study, which include BMI, socio-demographic factors, and PA, were determined only by means of self-administrated questionnaires, and they could be subject to bias. Self-reported results may cause an underestimation of weight status [23] and an inaccurate estimation of PA time as a result of recall bias. In addition, diet, which affects weight, was not assessed in the current study [24]. Finally, the present study had limited ability in obtaining representative samples because it relied on an Internet-based survey. The respondents to Internet-based surveys may possess certain characteristics, such as being younger, being more educated, having a higher income, having greater access to the Internet, and being more likely to respond to a survey if they are interested in the surveys content or are attracted by the incentives offered for participation [25,26]. Thus, the results of the present study may be less applicable to the general population, particularly among those with a lower level of education. Despite these considerations, the present study has some strengths. This study has a large sample size (n=1,420) and specifically focused on a sex subgroup with a higher prevalence of overweight (men). In addition, except for educational level, the distribution of age, marital status, job status, household income, prevalence of overweight, and attaining the total PA recommendations was similar to that for the general Japanese population [4,27,28]. Therefore, the findings of the current study could provide important implications in that the patterns and socio-demographic correlates of self-reported PA differed between normal-weight and overweight men. Future studies are needed to further identify correlates of PA by different BMI status in developing tailored PA intervention for the overweight population.

## Conclusions

The results revealed that patterns and socio-demographic correlates of self-reported PA in overweight men are different from those in normal-weight men. This finding suggests that it is necessary to develop specific strategies for PA intervention among overweight men. Socio-demographic correlates of self-reported PA may be more important for normal-weight than overweight men. To enhance the health of overweight men, it is important to further examine psychosocial and environmental correlates of PA in future studies.

## Competing interests

The authors declare that they have no competing interests.

## Authors contributions

YL contributed to the analysis and interpretation of the data and drafted and revised the paper. KH participated in the study design, contributed to the analysis and interpretation of data, and helped in drafting the manuscript. AS, KI, KO, and SI conceived of the study, participated in its design and coordination, and revised the paper. YN and TS performed the sequence alignment and revised the paper. All the authors have read and approved the final manuscript.

## Pre-publication history

The pre-publication history for this paper can be accessed here:

http://www.biomedcentral.com/1471-2458/12/278/prepub
